# Cluster randomized controlled trial of a multilevel physical activity intervention for older adults

**DOI:** 10.1186/s12966-018-0658-4

**Published:** 2018-04-02

**Authors:** Jacqueline Kerr, Dori Rosenberg, Rachel A. Millstein, Khalisa Bolling, Katie Crist, Michelle Takemoto, Suneeta Godbole, Kevin Moran, Loki Natarajan, Cynthia Castro-Sweet, David Buchner

**Affiliations:** 1 Department of Family Medicine & Public Health, University of California, San Diego, 9500 Gilman Drive, La Jolla, CA 92093 USA; 2 Group Health Research Institute, 1730 Minor Ave Suite 1600, Seattle, WA 98101 USA; 3 Harvard Medical School, 25 Shattuck St, Boston, MA 02115 USA; 4 Department of Preventive Medicine, Northwestern University, 680 N. Lake Shore Drive, Suite 1400, Chicago, IL 60611 USA; 5 Omada Health, Inc., 500 Sansome Street, San Francisco, CA 94111 USA; 6 University of Illinois at Urbana Champaign, 906 S. Goodwin Avenue, Urbana, IL 61801 USA

## Abstract

**Background:**

Older adults are the least active population group. Interventions in residential settings may support a multi-level approach to behavior change.

**Methods:**

In a cluster randomized control trial, 11 San Diego retirement communities were assigned to a physical activity (PA) intervention or a healthy aging attention control condition. Participants were 307 adults over 65 years old. The multilevel PA intervention was delivered with the assistance of peer leaders, who were trained older adult from the retirement communities. Intervention components included individual counseling & self-monitoring with pedometers, group education sessions, group walks, community advocacy and pedestrian community change projects. Intervention condition by time interactions were tested using generalized mixed effects regressions. The primary outcomes was accelerometer measured physical activity. Secondary outcomes were blood pressure and objectively measured physical functioning.

**Results:**

Over 70% of the sample were 80 years or older. PA significantly increased in the intervention condition (56 min of moderate-vigorous PA per week; 119 min of light PA) compared with the control condition and remained significantly higher across the 12 month study. Men and participants under 84 years old benefited most from the intervention. There was a significant decrease in systolic (*p* < .007) and diastolic (*p* < .02) blood pressure at 6 months. Physical functioning improved but the changes were not statistically significant.

**Conclusions:**

Intervention fidelity was high demonstrating feasibility. Changes in PA and blood pressure achieved were comparable to other studies with much younger participants. Men, in particular, avoided a year-long decline in PA.

**Trial registration:**

clincialtrials.gov Identifier: NCT01155011.

**Electronic supplementary material:**

The online version of this article (10.1186/s12966-018-0658-4) contains supplementary material, which is available to authorized users.

## Background

Physical inactivity has been identified as a major public health problem, particularly for older adults [1]. In addition to longevity, physical activity (PA) confers many health benefits to older adults including maintaining physical functioning and controlling conditions such as hypertension that are prevalent in this age group [2]. Several reviews have shown that PA in older adults can be increased in short term (6 month) controlled trials in multiple settings using behavioral and cognitive approaches at the individual or group level [3, 4]. In 2015, the Office of the Surgeon General in the US Department of Health and Human Services released “Step It Up! The Surgeon General's Call to Action to Promote Walking and Walkable Communities” to recognize walking as an important way to promote physical activity and researchers have proposed ecological approaches to reach larger population groups [5]. While there are many successful walking interventions [6], few interventions in older adults have included pedestrian advocacy to promote environmental changes [7] or assessed long-term sustainable approaches to behavior change.

Retirement communities provide opportunities and supportive environments for older adults’ PA, but residents are still not meeting PA guidelines [8]. Retirement communities, however, provide an ideal setting to study multi-level interventions as individual (goal setting), interpersonal (group walks) and environmental components (pedestrian advocacy to improve local walkability) can be delivered in and around a single location where a large vulnerable population resides [9, 10].

The primary objective of this study was to determine the effect of a 6 month multilevel PA intervention on objectively measured daily minutes of PA among older adults living in retirement communities. The secondary aim was to assess intervention effects on blood pressure and physical functioning. The exploratory aim was to assess intervention effects on self-reported physical and emotional outcomes. We explored demographic moderators of effects as other studies have found differences by age and gender [11]. We hypothesized that PA levels would increase in the intervention condition compared to the healthy aging attention control and be sustained over 12 months study and men and younger adults would benefit more from the intervention.

## Methods

### Study setting and population

The institutional review board approved the study; a Multilevel Intervention for Physical Activity in Retirement Communities (MIPARC) and the study was conducted according to the Helsinki declaration. An independent data and safety monitor reviewed all adverse events. All participants provided written informed consent.

MIPARC was a cluster randomized trial conducted in retirement communities in San Diego County. Sites had to house at least 100 residents, include independent living options, and have a store or park within 1 mile. Randomization was assigned at the site level. Both arms of the study were described to site managers and their written agreement to participate was procured prior to randomization. Once a memorandum of understanding was received, the statistician revealed the randomization condition for the next number on the list to the project manager, who informed the study site of which program they would receive. Peer leaders were then trained in the intervention they were allocated. Participants were recruited, unblended, to the specific study condition by peer leaders and no information about their health or behavior was available prior to randomization.

Older adult peer leaders, who helped deliver the program, were identified by site staff and residents. They were required to meet the same eligibility criteria as participants. Participants were recruited through presentations from UCSD staff, flyers, information tables, and peer leader outreach. Eligible participants had to be over 65 years old, complete a ‘timed up and go’ [12] in less than 30 s (for screening purposes only), be able to walk 20 m without human assistance, have not had a fall in the previous 12 months that resulted in hospitalization, be able to talk over the phone, and have no plans to move in the next year. Within sites, educational groups were limited to less than 35 participants to keep group activities manageable. This resulted in 7 sites with 9 educational groups in the control condition and 4 sites with 6 educational groups in the intervention condition. Thus an additional clustering level of education group (*N* = 15) was created post randomization. Participants were enrolled between 2010 and 2013 and followed through 2014.

### Intervention

The multilevel MIPARC intervention was described previously [13]. It employed techniques from the Social Cognitive Theory and applied them in an Ecological framework with intervention activities occurring at the individual (goal setting), interpersonal (group walks), and community level (pedestrian advocated improvements in walkability). Peer leaders helped deliver the program. Activities included up to 9 group education sessions (interpersonal level) over a 6 month period at sites. Group sessions aimed to provide information, social support, and behavior modeling. Group walks were co-led by UCSD staff and peer leaders for the first 6 months, with the peer leaders continuing this after 6 months. Four counseling phone calls were completed in the first 8 weeks to identify barriers and support safe goal setting (individual level). Participants wore pedometers and completed weekly step logs. The focus of the intervention was ‘Every Step Counts’, we did not require any specific intensity of PA. In the first 6 months of the study, their steps were plotted in progress charts to provide feedback every two weeks. Participants were encouraged to continue to wear the pedometer and return the logs to peer leaders in the second 6 months. All participants, regardless of baseline steps, were encouraged to achieve a daily 3000 step increase from their baseline over a period of 12 weeks, then focused on maintaining that increase for the remainder of the study. Weekly goal setting was discussed with the phone counselors and goal achievement was celebrated in the group sessions. Participants received educational materials, step counts for common locations around their campus, and walking maps for their local community. Peer leaders advocated for local environment improvements (environment level) to ensure participants had safe walking routes in their neighborhoods. They completed a walk audit with the community advocacy organization ‘Walk San Diego’ and received training to communicate with local policy makers and city officials. Community improvements achieved included extending crosswalk times and adding auditory and visual traffic cues at busy intersections, cleaning up a pedestrian access bridge, cutting back foliage that had grown over sidewalks, adding parking bollards to prevent cars parking over a sidewalk, and adding walking paths to a redevelopment plan. Changes to the environments occurred on average by 6 months but ranged from 3 to 12 months.

### Control

Participants in the healthy aging attention control condition received similar levels of attention on the same schedule as intervention participants. They received up to 9 group education sessions in the first 6 months on topics related to successful aging and completed 4 general health calls with UCSD staff counselors within the first 8 weeks.

### Outcomes

All participant assessments occurred at the retirement communities. The accelerometer data processor and statistician were blinded to the outcomes. In field assessors were not blinded as the intervention conditions were apparent at each site, for example only intervention participants had pedometers and visible celebration boards.

#### Primary outcome

Participants wore an accelerometer on a belt at the hip at baseline, 3, 6, 9 and 12 months for 6 days. Actigraph GT3X- plus data were aggregated to the minute level using the low frequency extension. Participants wore the device for waking hours only and non-wear time was excluded by the validated Choi algorithm in Actilife 6 with 90 consecutive minutes of zero counts, a 2 min tolerance and a 30 min small window [14]. Participant data were screened for a daily wear time of 10 h. We assessed multiple cut points for PA (see Additional file 1: Table S1) but focused on the most commonly used Freedson cut point (1952 counts per minute) for main analyses [15]. For older adults, any physical activity is considered beneficial but higher intensity activity may confer more benefits [2].

#### Objective secondary outcomes

At baseline, 6 and 12 months, participants completed the Short Physical Performance Battery (SPBB) [16] and timed 400 m walk test that results in a walking speed outcome [17]. Blood pressure was measured at rest using standard sphygmomanometer procedures. The mean of three measures was calculated.

#### Exploratory self-reported outcomes

Self-reported outcomes included the Centers for Epidemiologic Studies Depression scale, Perceived Quality of Life scale, Perceived Stress Scale, Fear Efficacy Scale International, PROMIS Pain Interference scale and the Late Life Function and Disability Instrument [18–23].

### Statistical analyses

An intention to treat analysis was conducted. The statistician was blinded to site condition. Generalized mixed effects regression methods were employed to assess intervention condition by time interaction. All models adjusted for site level clustering as appropriate for a cluster RCT. Partially complete records are included in the model, avoiding biases associated with a completers only analysis. This method gives unbiased estimates under the assumption that data are “missing at random” [24]. Analyses were conducted in R with educational group (*N* = 15 across the 11 sites) entered as a random clustering effect for all analyses. For accelerometer analyses, days were nested within participants, and a random participant-level intercept was included in the model. Condition (intervention versus control), time, and a two way interaction effect, condition x time, were included as fixed-effects. Models were adjusted for baseline differences in age, marital status, and physical functioning, as covariates. There was no difference in the adjusted and unadjusted models therefore the adjusted models are presented. Accelerometer wear time was entered as a fixed effect covariate in the primary outcome analyses. Three way interaction effects were explored to assess the moderating effects of demographic variables on the intervention.

The PA outcome demonstrated a negative binomial distribution, hence the generalized linear mixed model was applied with logarithmic link functions. A logistic regression mixed model with binomial link was used for the pain outcome; for all other health outcomes linear mixed effects models with Gaussian link were used. Fear of falling and depression scores were log transformed. A *p*-value of <.05 was employed for main effects and interactions.

For the primary outcome of accelerometer measured PA at 6 months, it was estimated that a sample size of 250 would provide a minimum of 80% power to detect a medium effect size (0.5 SD) accounting for an ICC of .07 for site cluster.

## Results

**Fig. 1:**
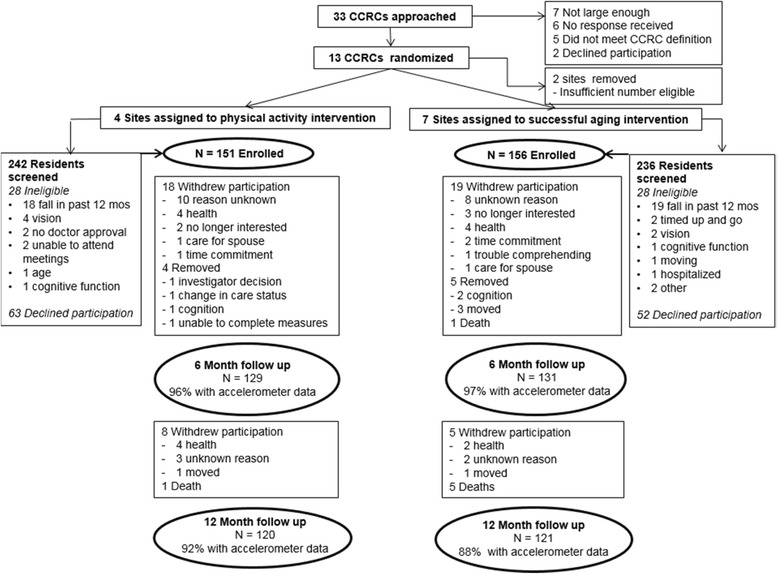
CONSORT diagram

Figure 1 provides a CONSORT flow diagram of recruitment, screening and enrollment; 73% of all participants who were eligible were enrolled. At 12 months, accelerometer data that met validity criteria were available for 92% of intervention participants and 88% of comparison participants. There were 7 sites randomized to control and 4 sites randomized to intervention. Within the control sites 9 educational groups were formed and in the intervention 6 educational groups were formed. Site level clusters ranged from 15 to 79 participants and educational groups within sites ranged from 15 to 30. Drop out varied by 11–47% by site level cluster. Intervention and controls had similar drop outs. The site level clustering ICC was .008. Table 1 presents descriptive statistics at baseline, adjusting for site level clustering. Participants in the intervention condition were significantly more likely to be younger and married and more likely to complete the 400 m walk. Participants represented the communities from which they were drawn, except that a greater percentage of males were enrolled compared to the population in the retirement community (i.e. 28% in our study compared to 20% in the communities). Baseline differences in characteristics were adjusted for in the mixed model approach and results did not differ between the adjusted and unadjusted models.

**Table 1:** Descriptive statistics for the sample at baseline at the participant level

	Control *N* = 156	Intervention *N* = 151	*P* value*
Mean age (years)	85.3 (6.5)	81.9 (5.9)	0.007
Gender (% female)	71	74	0.47
Education (% college and above)	60	69	0.11
Marital Status (% married)	30	52	0.0002
Moderate-Vigorous Physical Activity^a^	2.3 (1.8, 3.8)	3.1 (1.9, 4.9)	0.261
400 m walking speed (meters/s)	0.89(0.22)	1.00(0.21)	0.02
SPPB score ^b^	7.96 (2.79)	9.25 (2.60)	0.07
Systolic BP ^c^	130.70 (19.07)	132.06 (19.24)	0.59
Diastolic BP ^c^	67.00 (8.88)	69.24 (11.18)	0.81
% with BP ^c^ over 150/90	13.73	21.19	0.48
LLFDI ^d^ score (range 10–50)	36.34 (8.94)	40.54 (8.32)	0.29
QoL ^e^ (range 1–5)	3.85 (0.64)	4.00 (0.64)	0.26
CESD ^f^ (range 0–30)	6.17 (4.24)	4.80 (3.76)	0.19
Stress (range 0–16)	4.60 (2.51)	3.79 (2.47)	0.08
Pain (range 6–30)	10.02 (4.50)	9.02 (4.25)	0.41
Fear of falling (range 16–64)	27.88 (9.20)	23.92 (6.89)	0.12

*Computed using general linear mixed models with adjustment for site level clustering as a random effect

^a^Negative Binomial mixed model estimate with a ± 1 standard error adjusting for nesting of days within people

^b^Short Physical Performance Battery

^c^Blood pressure

^d^Late Life Function and Disability Instrument

^e^Quality of Life

^f^Centers for Epidemiologic Studies Depression scale

### Primary outcome

**Fig. 2:**
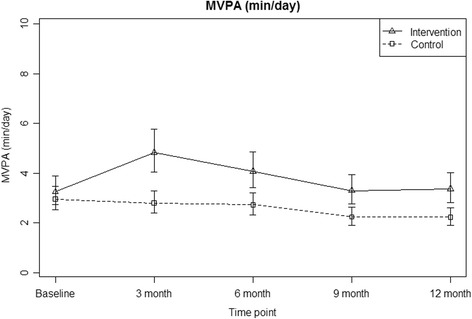
Differences in moderate vigorous physical activity (MVPA) between intervention and control conditions over time, adjusting for baseline demographic differences, nesting of days within people and people within sites

Table 2 presents the unadjusted minutes for multiple accelerometer cut points over time by the intervention conditions. In the moderate vigorous intensity category there was an 8 min increase in physical activity per day, for the lighter intensity category (e.g. 760 counts per minute) increases in physical activity were up to 17 min a day i.e. 119 min per week. Figure 2 presents the model based estimates of daily accelerometer moderate-vigorous PA minutes over the 12 month intervention period adjusting for baseline differences in demographics and physical functioning, accelerometer wear time, nesting of days within people and people within sites. Differences between the intervention and control conditions remained significant at 12 months.

**Table 2:** Descriptive statistics of the accelerometer data collected across all time points

	Sample N	Total number of days available across sample	Average (SD) daily wear time minutes per person	Average (SD) wear days per person	Average (SD) daily minutes 760 CPM and above per person	Average (SD) daily minutes 1041 CPM and above (SD) per person	Average (SD) daily minutes 1952 CPM and above (SD) per person^a^	Total daily CPM
Baseline								
Control	156	925	821.80 (81.70)	5.90 (1.60)	39.19 (28.30)	24.12 (20.72)	6.76 (10.28)	119,172.00 (61,583.76)
Intervention	151	836	805.10 (79.10)	5.50 (1.60)	50.03 (29.02)	31.74 (23.10)	10.53 (13.58)	141,578.8 (64,860.07)
3 Months								
Control	137	699	809.90 (81.30)	5.10 (1.40)	39.66 (29.49)	24.80 (21.94)	6.54 (9.91)	117,389.50 (61,771.74)
Intervention	128	666	825.10 (85.40)	5.20 (1.40)	67.24 (41.54)	46.02 (34.23)	18.31 (22.58)	178,941.30 (96,481.19)
6 Months								
Control	127	681	811.40 (86.70)	5.40 (1.20)	39.24 (25.11)	23.78 (18.08)	6.29 (8.56)	116,880.30 (52,371.70)
Intervention	124	648	821.50 (78.40)	5.20 (1.20)	59.46 (40.05)	40.59 (32.87)	15.60 (20.11)	162,962.20 (90,132.36)
9 Months								
Control	118	617	807.00 (89.50)	5.23 (1.47)	38.12 (26.53)	22.52 (18.32)	5.40 (7.83)	113,715.40 (54,800.43)
Intervention	116	613	818.53 (87.90)	5.28 (1.43)	53.56 (31.44)	35.46 (26.34)	12.87 (17.06)	152,992.00 (77,138.23)
12 Months								
Control	107	596	807.01 (89.47)	5.57 (1.30)	38.70 (26.50)	23.21 (19.62)	5.96 (9.68)	115,141.60 (56,778.73)
Intervention	110	602	816.58 (87.59)	5.47 (1.24)	56.79 (38.32)	37.45 (30.38)	13.38 (16.87)	156,379.70 (84,016.68)

^a^Statistical analyses for intervention effects presented for 1952 CPM only

Demographic moderators were examined as they have been found to be significant in previous studies [11]. The statistical analyses also revealed interactions by gender, age, marital status and education (*p* < .001). The largest interaction effects were for gender (see Additional file 2: Figure S1). Men almost doubled their PA levels from baseline in the intervention condition while men in the control condition greatly decreased their PA levels. The women did not have as large an increase or decrease in behavior across the two conditions. Participants less than 84 years old (the mean for this population) had greater increases in PA compared to control participants. Married participants in the intervention condition increased their PA more than those in the control condition who were married. Likewise college educated participants in the intervention condition increased their PA more than college educated participants in the control condition.

### Secondary outcomes

There was a significant time x condition interaction for both systolic (t value = − 2.68, *p* = .007) and diastolic (t value = − 2.35 *p* = .02) blood pressure at 6 months. Systolic blood pressure reduced by 6.8 (SD = 3.2) points on average, diastolic by 2.5 (SD = 1.9) points, and having clinically high blood pressure reduced by 18.2% (10.4%, 24.3%). The intervention effects were no longer significant at 12 months. There was a 0.02 m/s (SD = 0.03) improvement in walk speed and 0.26 (0.28) improvement in SPPB score between baseline and 6 months but the differences were not statistically significant.

None of the exploratory survey outcomes significantly increased in the intervention condition over time. Table S1 shows the unadjusted change in these survey measures over time.

In total, 174 adverse events were reported during the 12 month study period, 68 of which met our criteria for serious adverse events (SAE), based on NIH guidelines. The SAEs by person are reported in Additional file 3: Table S2. No SAEs were determined to be definitely related to study participation and there was no significant difference in the number of events, including falls, between the intervention and control conditions, although physical injuries were higher in the intervention group.

### Fidelity

Attendance at the group education sessions, assessed by staff checking from a list of names, was 82% on average ranging from 93 to 76% in the intervention condition over the 6 months and from 87 to 73% in the control condition. The completion rate of phone counseling calls with participants (logged by callers and verified by recording) was 90% on average. In the intervention condition daily enactment of intervention was high. On average 96% met a daily increase of 1000 steps, 78% met a daily increase of 2000 steps and 51% met a daily increase of 3000 steps assessed by returned pedometer logs entered by staff.

## Discussion

A significant increase in PA was found for the intervention; about 56 min of moderate-vigorous PA per week or 119 min of light PA, compared to no change overall in the controls. The difference remained significant over the 12 month intervention period, although the greatest increase was in the first 3 months. The guidelines for older adults encourage any increase in physical activity as beneficial, and increases in moderate-vigorous PA as more beneficial [2]. Both light and moderate PA improved in this study, despite the focus on steps rather than intensity. Some subgroups, men and younger participants, benefited from this intervention more than others. Adverse events were similar to other PA intervention trials [25], even though participants were much older than other studies and intervention participants were encouraged to walk in local neighborhoods, if it was safe to do so. Intervention adherence was high. Our findings for PA were similar to other studies in older adults [25–29], even though our sample was much older.

The impact of the intervention on blood pressure had clinical significance with a reduction of 7 points in systolic blood pressure. The weekly increase in PA minutes could contribute to participants meeting PA guidelines and having reduced risk of cancer, heart disease, and diabetes [2]. There was no impact of the intervention on self-reported outcomes or the physical functioning measures. This could have been due to high rates of functioning at baseline, lack of statistical power, or because the walking intervention was insufficient to impact these outcomes.

In men, this intervention prevented a 12 month decline in PA of 45%. MIPARC included male peer leaders as role models and allowed for independent walking and encouraged neighborhood walking, which may have appealed to men more than group exercise classes typically offered at sites. These positive effects for men are important in light of recent findings that men decrease their activity levels as they age [30]. MIPARC also successfully recruited older men, who are often not reached in PA programs [31].

While differences between the intervention and control conditions remained significant at 12 months, initial large increases in PA at 3 months were not maintained throughout. Many previous studies also demonstrate only short-term effects of PA interventions, but some studies (even simple pedometer interventions) have been successful over 6 months [6]. Few studies have assessed PA objectively through 12 months, especially in this much older age group [32].

The decline over time in some control participants, despite participants living in well facilitated and serviced retirement communities, indicates that supportive environments alone are insufficient for some groups e.g. men.

The steady decline in PA in the intervention group after 3 months is somewhat surprising given intervention activities continued and high attendance at events was maintained through 6 months. Further, the peer leaders continued events, including group walks and community advocacy, and participants continued to wear and return pedometer logs through 12 months. Our goal setting schedule, however, emphasized improvements should be achieved by 3 months and thereafter maintained. This could have led participants to focus too much on this short-term goal. Personal counseling phone calls also only occurred in the first 8 weeks of the study. These calls may have been important in this sample and previous studies have shown that PA counseling is effective [33]. Feasible and affordable ways to continue such personal support are needed. Training of peer leaders in such counseling techniques may be one solution [34].

Strengths of this intervention include the objective measures of PA, the multilevel approach to the intervention including peer leaders and community advocacy, and the retirement community setting that enabled us to reach much older adults than are typically included in PA trials. Further, this community setting holds promise for future implementation and dissemination efforts [35]. The study demonstrated that all levels of the ecological model could be targeted and high attendance suggests participants were not burdened by the multiple components. Peers were able to help deliver the interpersonal elements such as group activities. Local environmental changes related to walkability such as crossing timings and improving sidewalk access were achieved through peer led pedestrian advocacy efforts. The study design, however, did not allow us to assess whether any one component was more effective than another and high levels of fidelity across all levels do not support finding dose effects.

Limitations include the homogeneity of the participants. It is not clear if the current intervention would generalize to all older adults, especially since San Diego County has a temperate climate. Other limitations include significant differences in age, marital status and physical functioning between the two intervention conditions at baseline. Such imbalances are common in cluster RCTs and can only be controlled for if baseline characteristics are available to researchers before enrollment, such as patient data in a clinical setting [36]. This information was not available in the retirement community setting. Results were the same with and without adjustment for these differences. Further differences in the sample would have reduced our power and results for PA and blood pressure were significant. Such differences may have reduced significance for the physical functioning outcomes which showed similar rates of improvement as other PA trials [17]. Another imbalance that could have affected power was between the intervention and control sites (4 to 7) although the number of participants between intervention and control was almost equal and drop out did not differ by study arm. The difference in number of sites, however, across intervention and control conditions was due to no upper limit on the size of sites and agreements with sites that allowed all residents who were eligible to be enrolled. This resulted in a large intervention site that provided 3 educational groups within the site. We adjusted for educational group clustering in our analyses to handle this difference. Regardless of size all sites had similar recruitment rates (10%). In ability to blind assessors to intervention condition could also have affected findings, but given measures such as the physical functioning tests were not different between conditions it suggests there was no bias in the assessments.

## Conclusion

This study provides a model of training and multilevel intervention delivery that could be applied to other community settings. The multilevel PA intervention was acceptable to participants and peer leaders with high adherence rates. Men benefited most from the program. The increases in PA (up to 119 min light PA and 56 min moderate-vigorous PA per week) can contribute to older adults meeting daily PA guidelines. The intervention, despite including lower intensity activities, had a significant impact on blood pressure and hypertension rates. Efforts to maintain increases in PA in this age group, including personal counseling, are still needed.

## Additional files

Additional file 1: **Table S1.** Changes in exploratory outcomes overtime by intervention and control. (DOCX 16 kb)

Additional file 2: **Figure S1.** Gender differences in physical activity between intervention and control conditions over time, adjusting for baseline demographic differences, nesting of days within people and people within sites. (DOCX 17 kb)

Additional file 3: **Table S2.** Adverse events by condition at 12 months. (DOCX 14 kb)
