# Supplementary material for: Cluster randomized controlled trial of a multilevel physical activity intervention for older adults
Source: Int J Behav Nutr Phys Act. 2018 Apr 2;15:32. doi: 10.1186/s12966-018-0658-4 (PMC5879834; doi:10.1186/s12966-018-0658-4)
Supplement: Supplementary file 1 — Table S1. Changes in exploratory outcomes overtime by intervention and control. (DOCX 16 kb) [file 12966_2018_658_MOESM1_ESM.docx]

Table S1: Changes in exploratory outcomes by condition.

|  | Change in scores between timepoints | Intervention |  | Control |  |
| --- | --- | --- | --- | --- | --- |
|  |  | N | Mean (SD) | N | Mean (SD) |
| CESD ^a^ | ∆ 6M-BL | 113 | 0.3 (3.3) | 120 | 0.2 (3.3) |
|  | ∆ 12M-BL | 109 | 0.4 (3.7) | 105 | 0.6 (4.0) |
| QoL ^b^ | ∆ 6M-BL | 118 | 0.1 (0.5) | 122 | 0.0 (0.5) |
|  | ∆ 12M-BL | 113 | 0.0 (0.5) | 107 | -0.1 (0.6) |
| PSS ^c^ | ∆ 6M-BL | 118 | -0.3 (2.8) | 122 | 0.0 (2.8) |
|  | ∆ 12M-BL | 113 | -0.1 (2.8) | 107 | 0.0 (2.9) |
| FESI ^d^ | ∆ 6M-BL | 107 | 1.0 (7.9) | 112 | 1.8 (8.4) |
|  | ∆ 12M-BL | 102 | -2.0 (7.3) | 93 | -5.1 (8.7) |
| Pain ^e^ | ∆ 6M-BL | 115 | 1.2 (7.8) | 123 | 0.6 (6.8) |
|  | ∆ 12M-BL | 110 | 2.6 (7.7) | 107 | 0.5 (7.8) |
| LLFDI ^f^ | ∆ 6M-BL | 106 | -0.1 (3.4) | 115 | -1.3 (5.2) |
|  | ∆ 12M-BL | 104 | -1.3 (4.1) | 102 | -1.3 (4.4) |
|  | ∆ 12M-BL | 104 | -1.3 (4.1) | 102 | -1.3 (4.4) |

^a^ Centers for Epidemiologic Studies Depression scale; ^b^ Perceived Quality of Life scale, ^c^ Perceived Stress Scale; ^d^ Fear Efficacy Scale International; ^e^ PROMIS Pain Interference scale ^f^ Late Life Function and Disability Instrument;
