# Supplementary material for: Cluster randomized controlled trial of a multilevel physical activity intervention for older adults
Source: Int J Behav Nutr Phys Act. 2018 Apr 2;15:32. doi: 10.1186/s12966-018-0658-4 (PMC5879834; doi:10.1186/s12966-018-0658-4)
Supplement: Supplementary file 2 — Figure S1. Gender differences in physical activity between intervention and control conditions over time, adjusting for baseline demographic differences, nesting of days within people and people within sites. (DOCX 17 kb) [file 12966_2018_658_MOESM2_ESM.docx]

Figure S1. Gender differences in physical activity between intervention and control conditions over time, adjusting for baseline demographic differences, nesting of days within people and people within sites
