# Supplementary material for: Cluster randomized controlled trial of a multilevel physical activity intervention for older adults
Source: Int J Behav Nutr Phys Act. 2018 Apr 2;15:32. doi: 10.1186/s12966-018-0658-4 (PMC5879834; doi:10.1186/s12966-018-0658-4)
Supplement: Supplementary file 3 — Table S2. Adverse events by condition at 12 months. (DOCX 14 kb) [file 12966_2018_658_MOESM3_ESM.docx]

Table S2. Adverse events by condition at 12 months

| ***Serious Adverse Events*** | **Intervention**  **N=151**  **# of people (%)** | **Control**  **N=156**  **# of people (%)** | **p value** | |
| --- | --- | --- | --- | --- |
| Death | 1 (0%) | 5 (3%) | 0.19 |  |
| Fall | 20 (13%) | 16 (10%) | 0.42 |  |
| Serious physical injury (e.g. broken bone, stress fracture, joint injury) | 5 (3%) | 1 (1%) | 0.10 |  |
| Persistent or significant physical or cognitive disability or incapacity (e.g. stroke, cancer, cognitive disability) | 3 (2%) | 3 (2%) | 0.97 |  |
| Overnight hospitalization for any reason | 10 (7%) | 5 (3%) | 0.17 |  |
